# Parathyroid Carcinoma All-In-One, a Rare Life-Threatening Case With Multiple Systemic Manifestations: Case Report and Review of the Literature

**DOI:** 10.3389/fendo.2022.881225

**Published:** 2022-07-07

**Authors:** Lorenzo Zelano, Pietro Locantore, Carlo Antonio Rota, Caterina Policola, Andrea Corsello, Esther Diana Rossi, Vittoria Rufini, Luca Zagaria, Marco Raffaelli, Alfredo Pontecorvi

**Affiliations:** ^1^ Department of Translational Medicine and Surgery, Unit of Endocrinology, Università Cattolica del Sacro Cuore—Fondazione Policlinico “Gemelli” IRCCS, Rome, Italy; ^2^ Institute of Pathology, Università Cattolica del Sacro Cuore—Fondazione Policlinico “Gemelli” IRCCS, Rome, Italy; ^3^ Institute of Nuclear Medicine, Università Cattolica del Sacro Cuore—Fondazione Policlinico “Gemelli” IRCCS, Rome, Italy; ^4^ Department of Endocrine and Metabolic Surgery, Università Cattolica del Sacro Cuore—Fondazione Policlinico “Gemelli” IRCCS, Rome, Italy

**Keywords:** parathyroid carcinoma, hypercalcemia, brown tumors, hungry bone, pancreatitis, venous thrombosis, synovitis, Neuropathy

## Abstract

Parathyroid carcinoma (PC) is an extremely rare disease. Although it may occasionally occur in genetic syndromes, it is more often sporadic. It is usually associated with a consistent secretion of PTH, causing severe hypercalcemia and potentially all clinical conditions due to primary hyperparathyroidism. Management of PC can be challenging: some clinical, biochemical, and radiological features may be useful, but the final diagnosis of malignancy strictly relies on histological criteria. To date, radical surgery is the first-choice treatment and is the only effective therapy to control hypercalcemia and other clinical manifestations. On the other hand, chemo- or radiotherapy, local treatments, or novel drugs should be reserved for selected cases. We report an exceptionally unusual case of life-threatening PC, associated with several systemic manifestations: moderate pancreatitis, portal thrombosis, kidney stones, brown tumors, osteoporosis, hungry bone syndrome (HBS), chondrocalcinosis, neuropathy, and depression. The clinical case also represents an opportunity to provide a review of the recent literature, associated with a complete evaluation of the main diagnostic and therapeutic approaches.

## 1 Introduction

Parathyroid carcinoma (PC) is an extremely rare malignancy. Since 1909, when first discovered by de Quervain, the number of cases reported in the literature still does not exceed one thousand. In 1969, Holmes et al. (1) reviewed the data of the first 46 cases of PC published since the original description of the disease. They found that subjects affected by PC generally presented marked and symptomatic hypercalcemia as well as skeletal and renal complications of hyperparathyroidism. These observations suggested that parathyroid malignant disease usually has a different clinical pattern than the much more common benign form of primary hyperparathyroidism. 

Because of its rarity, a standardized diagnostic, prognostic, and therapeutic approach has not been provided yet and the TNM staging algorithm is not universally accepted (2). Most patients show a sporadic form, whereas some genetic syndromes may be occasionally associated with PC, such as hyperparathyroidism jaw-tumor syndrome (HPT-JT), multiple endocrine neoplasia (MEN) syndromes type 1 and type 2A, as well as isolated familial hyperparathyroidism. Anyway, no case of transformation from benign parathyroid disorder to malignancy has been documented, even if synchronous parathyroid adenoma (PA) and carcinoma can be found (3).

In more than 90% of cases, the presence of a parathyroid malignancy is associated with a consistent secretion of PTH, causing hypercalcemia and potentially all clinical conditions due to primary hyperparathyroidism, although non-secreting parathyroid carcinoma can occur. The most common findings are gastrointestinal symptoms, such as bloating, constipation, peptic ulcer, or pancreatitis; kidney manifestations with nephrolithiasis, hypercalciuria, or reduced glomerular filtration rate; skeletal dysfunction with osteoporosis or fragility fracture; neurocognitive dysfunction; cardiovascular disease (4). In 1982, Shane and Bilezikian (5), reviewing all 62 cases of PC reported in the English literature during the previous 12 years, outlined a well-defined clinical description of PC, in contrast with benign parathyroid lesions. Usually, the patient with PC is younger with no difference in prevalence between male or female; moderate to severe hypercalcemia and target organ involvement is common, particularly with the simultaneous presence of both bone and stone disease; finally, a palpable neck mass is more likely to be found in case of malignancy.

## 2 Case Report

### 2.1 Pre-Operative Findings

A 56-year-old man presented in the emergency department complaining of progressive fatigue, severe abdominal pain, vomiting, dehydration, and weight loss of almost 10 Kg in the previous 6 months.

Since blood exams showed corrected calcium values of 17.9 mg/dl, associated with high parathormone (PTH) levels (1518 pg/ml) and low phosphorus levels (0.8 mg/dl), medical therapy was started with profuse hydration (3000 ml IV/day), diuretics (furosemide 20 mg IV tid), and bisphosphonates (zoledronic acid 4mg IV once).

Contrast-enhanced CT scan performed in another hospital showed several systemic manifestations: multiple heterogeneous abdominal lesions, as for past moderate pancreatitis with peripancreatic fluid collection; flow void in the portal vein, as for venous thrombosis; bilateral medullary nephrolithiasis; multiple osteolytic lesions, spread over the whole bone, the larger of which located in the left iliac bone. Low molecular weight heparin (LMWH) and intravenous antibiotic therapy were administered to contrast portal thrombosis and the infective risk associated with pancreatitis.

**Figure 1:**
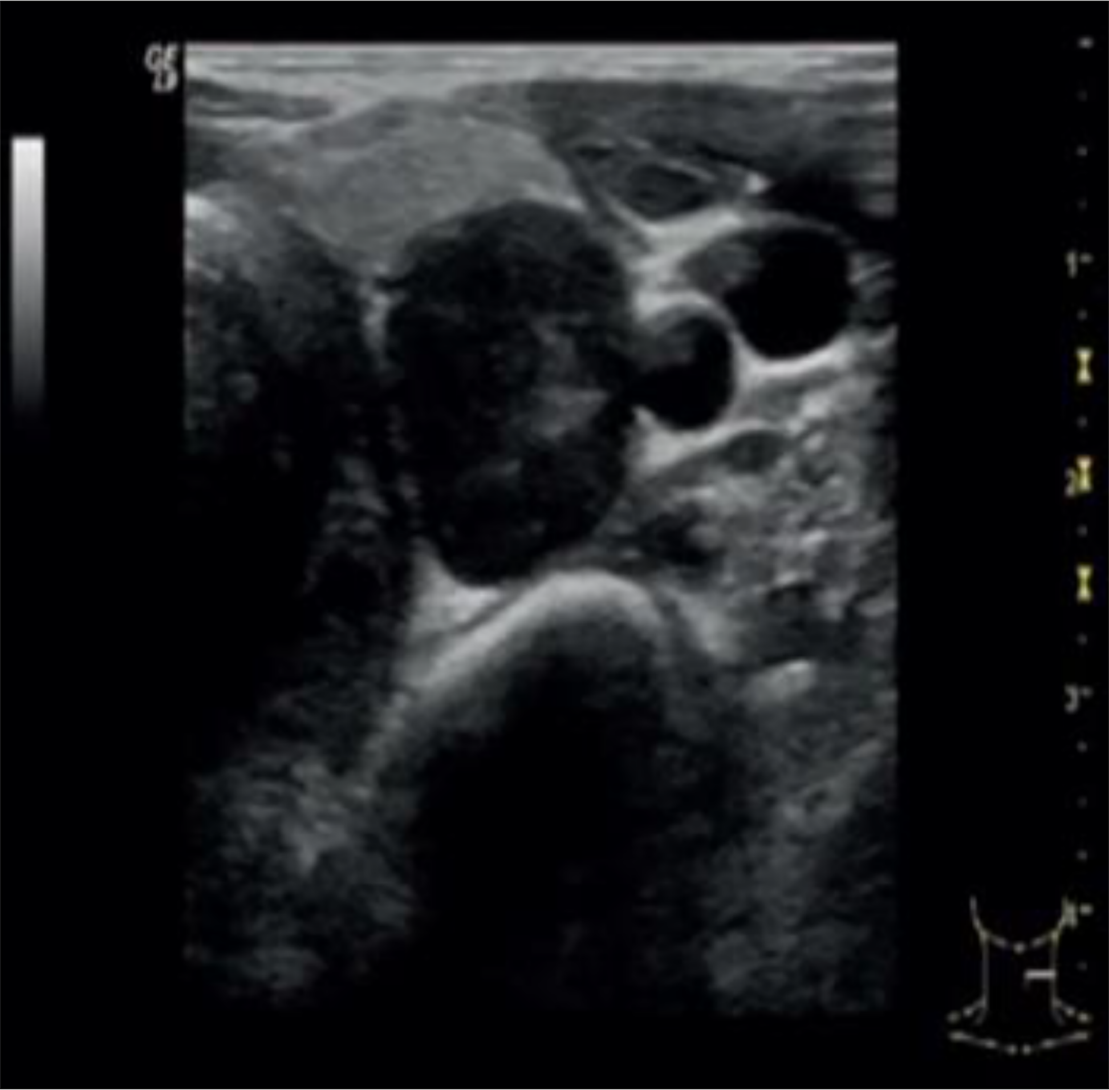
Neck ultrasound showed a 3.6 cm round hypoechoic nodule posterior to the left inferior thyroid lobe with undefined margins; no signs of infiltration of local tissues were detected.

**Figure 2:**
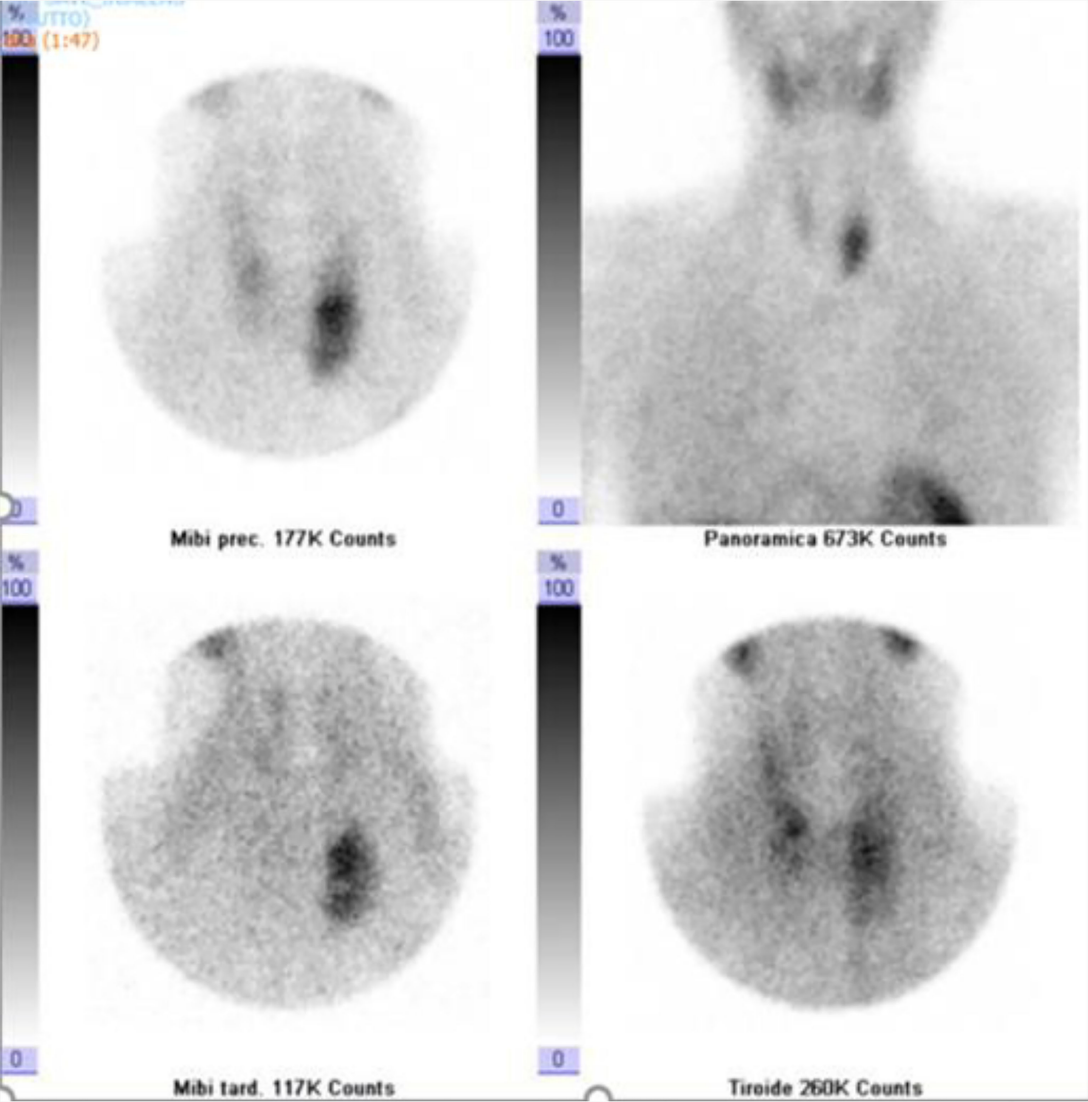
Dual-phase Tc-99m sestamibi scintigraphy. Early (5 and 30 min.) and delayed (60 and 120 min.) images showed a persistent accumulation of radiopharmaceuticals in the left inferior parathyroid region.

Neck ultrasound showed a 3.6 cm round hypoechoic nodule posterior to the left inferior thyroid lobe with undefined margins; Colour Doppler showed increased vascularity in the nodule (**Figure 1**). The same lesion was detected by Technetium-99m MIBI scan, documenting a persistent uptake in the left inferior parathyroid region (**Figure 2**).

**Figure 3:**
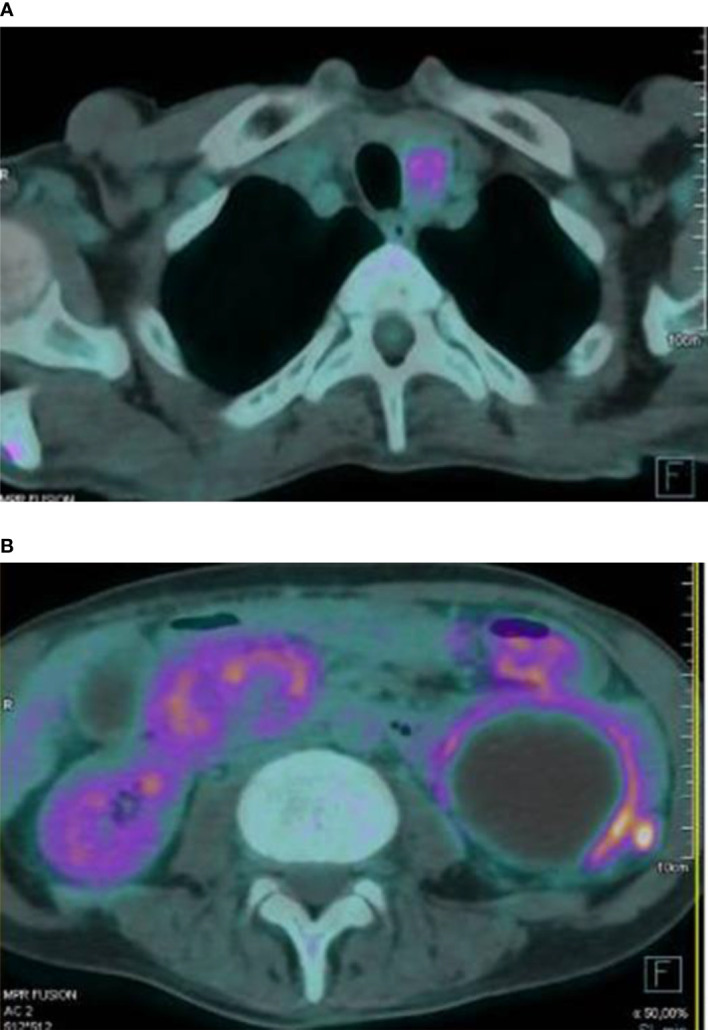
Intense glucose uptake in the anterior neck region was observed at 18-FDG PET/CT scan **(A)**. The exam also documented a peripheral mild glucose uptake from several abdominal masses **(B)**.

Furthermore, 18-FDG PET/CT showed mild glucose uptake from abdominal masses, suspicious of inflammatory processes; diffuse glucose uptake from skeletal lesions; and intense homogenous uptake in the anterior neck region, with SUVmax value of 4.8 (**Figure 3**). To rule out malignancy, skeletal biopsy of the iliac lesion was performed, showing giant cell lesions histologically traceable as brown tumor.

Pre-operatory screening was completed by DEXA scan, suggestive of severe lumbar and femoral osteoporosis (lumbar and femoral neck T- score of -3.2 and – 3, respectively). To rule out other endocrinopathies, serum calcitonin, prolactin, chromogranin A, and urinary catecholamine test was performed and resulted in range.

### 2.2 Surgery and Histopathology

During surgery, a hard 4 cm mass with undefined margins was found, infiltrating the lower segment of the left thyroid lobe. For that left inferior selective parathyroidectomy was performed, including en bloc resection of the left thyroid lobe, thymectomy, and central neck lymph nodes dissection.

Histology showed a PC and confirmed that the left thyroid lobe was infiltrated, while all cervical lymph nodes were not involved (pT2, N0 according to TNM/AJCC 8TH ed. 2017). Microscopic examination showed that the parathyroid lesion had a solid structure, with fibrous trabeculae, mainly composed of principal cells with nuclear dysmorphism and irregularities. The presence of haemorrhagic component and focal necrosis was detected. About 5 mitoses for 10 hpf were observed. Immunohistochemical stains showed positivity for GATA-3, PTH, Galectin-3, while negativity for TTF1 and Thyroglobulin. The ki-67 was 5%.

The need for adjuvant radiotherapy was fully discussed during a multidisciplinary board, composed by endocrinologist, oncologist, radiologist, pathologist, surgeon, and radiotherapist. Based on the low tumoral stage at diagnosis and the current literature about radiotherapy in PC (6), active surveillance was recommended, with neck ultrasound imaging and biochemical assays, reserving further treatment in case of recurrence of disease.

### 2.3 Post-Operative Course

PTH and calcium levels declined after surgery. Particularly, intraoperative-PTH dropped from 1643 to 163 pg/ml; serum PTH lowered from 1518 pg/mL to 89 pg/mL the day after surgery; also, serum calcium passed from 14.4 mg/dl on the day of surgery to 12.3 mg/dl 24 hours later. Then the value of calcium stably remained around 10 mg/dl during the three following days, for that no calcium supplementation was introduced at discharge, mistakenly. Indeed, calcium levels went progressively down so that the patient experienced symptomatic hypocalcemia (calcium value of 6.7 mg/dl) two weeks after surgery, due to HBS. Therefore, calcitriol (0.5 mcg tid) and calcium gluconate therapy (3000 mg IV/day) were started, with a progressive decrease in the calcium administration.

**Figure 4:**
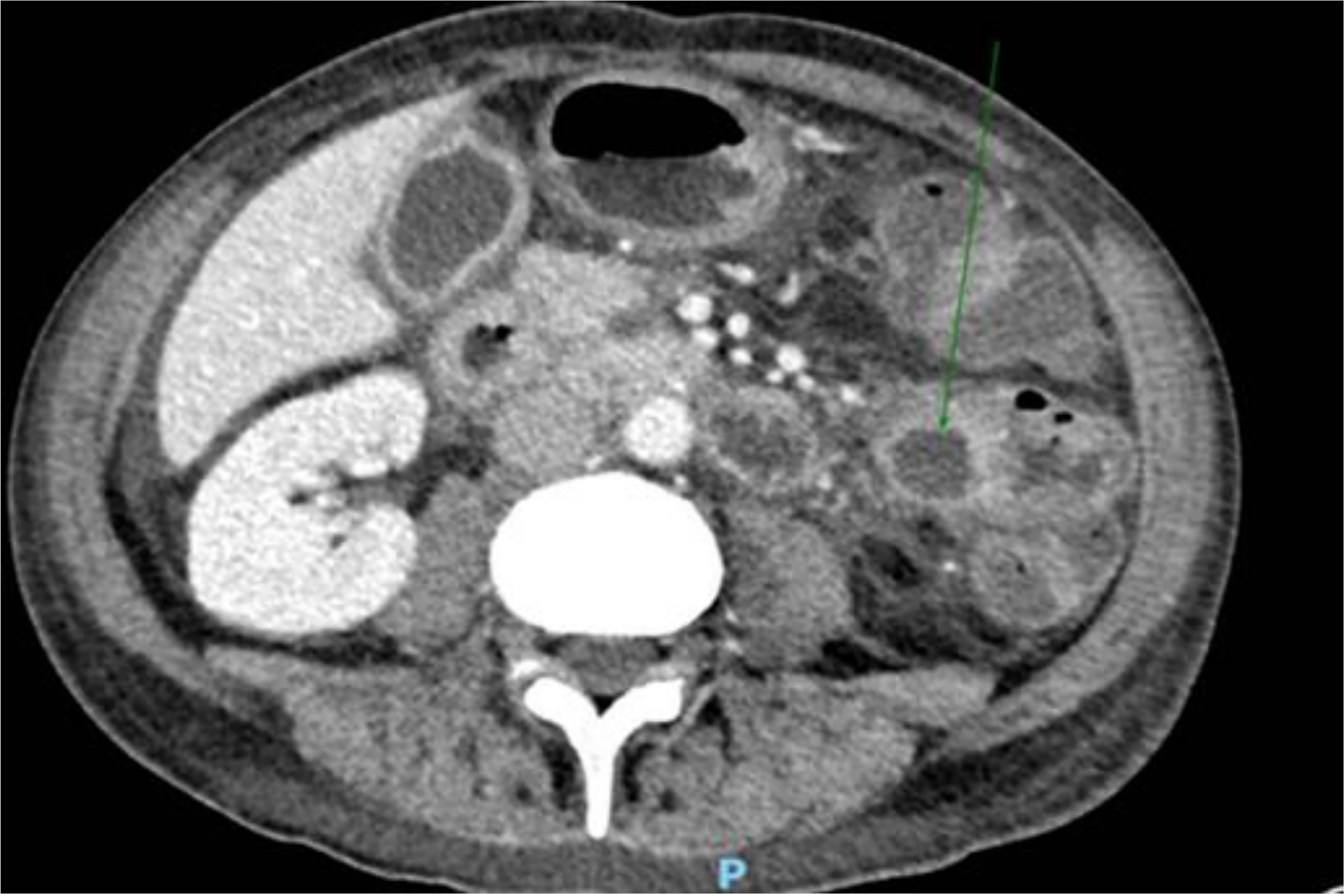
Consistent reduction of the inhomogeneous abdominal masses, as for physiological evolution of pancreatitis in resolution, documented at CT scan.

To treat the portal thromboembolism, LMWH was continued for 2 months when the CT scan showed a partial resolution of portal thrombosis. The CT scan also showed a consistent reduction of the inhomogeneous abdominal masses, as for physiological evolution of pancreatitis in resolution (**Figure 4**). The level of serum amylase declined from 432 UI/ml to 182 UI/ml after about 2 weeks. Nevertheless, the patient developed pancreatic exocrine insufficiency and he had to assume pancreatic enzyme replacement therapy 3 times a day for 6 months after diagnosis. On the other hand, nephrolithiasis remained asymptomatic and renal impairment did not occur. After 2 months, anticoagulant therapy with fondaparinux was started with the indication of a radiologic follow-up after 4 months, which showed a complete resolution of the thrombosis.

**Figure 5:**
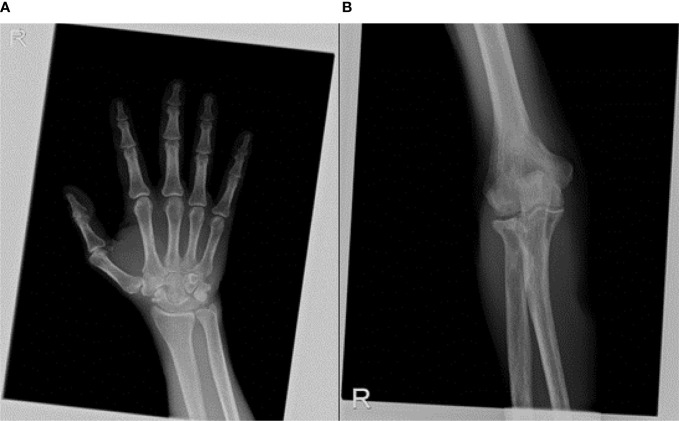
X-ray of articular sites, such as wrist **(A)** and elbow **(B)** showed a cartilaginous deposit of calcium pyrophosphate, as for chondrocalcinosis.

A rheumatologic evaluation was also performed during hospitalization, since the patient also experienced a widespread articular pain, which started 2 months before the diagnosis and progressively worsened, regardless of the treatments used. Ultrasound articular imaging of hands, wrists, elbows, knees, and ankles showed the presence of calcification in the cartilage around the joints, associated with synovitis; X-ray of the same sites confirmed a cartilaginous deposit of calcium pyrophosphate, as for chondrocalcinosis (**Figure 5**). Biochemical assays for autoimmunity (rheumatoid factors, ACPA, ANA, ENA, ANCA) resulted negative, while CRP remained high even after resolution of the abdominal infection. Oral colchicine was started, associated with 25 mg prednisolone. Since rapid clinical improvement was reached, corticosteroids were rapidly reduced and then interrupted after 3 weeks, while chronic therapy with 1 mg colchicine was confirmed.

Moreover, the patient experienced depression and a spread neuromuscular pain, described as burning and tingling for several weeks, even if serum calcium was fully normalized. Electromyography documented a peripheral polyneuropathy. Since both mood disorder and neurological disease had a consistent improvement after surgery and nutrient supplementation, no specific medication was started.

Calcium therapy was first progressively reduced and then interrupted after 4 months, while calcitriol was reduced to 0.5 mcg a day. It was suspended 8 months after surgery when biochemical assay showed that PTH and calcium values were in range (**Table 1**) and neck ultrasound documented no recurrence of the disease.

**Table 1:** Main biochemistries before and after parathyroid surgery.

	Pre-operative	Post-operative (1 day)	Post-operative (2 weeks)	Post-operative (4 months)	Post-operative (8 months)
**Parathyroid Hormone** **(14-72 pg/ml)**	1518	89	72.4	87	38.3
**Serum Calcium** **(8.6 -10.2 mg/dl)**	17.9	12.3	6.7	8.6	9.6
**Serum Phosphate** **(2.5-4-5 mg/dl)**	0.9	1.1	1.9	2.8	3.1
**Creatinine** **(0.67-1.17 mg/dl)**	1.08	–	0.92	0.67	0.74
**Alkaline Phosphatase** **(40-129 UI/L)**	273	-	234	161	118
**Treatment**	Hydration (3 L IV/day) Furosemide (20 mg tid) Zoledronate (4 mg IV once)	–	Calcitriol (From 0.5 mcg tid) Calcium (From 3 g/day)	Calcitriol (0.5 mcg/day)	–

Concomitant medications to treat hyper- and hypocalcemia are reported.

## 3 Discussion

### 3.1 Diagnosis

PC is a very rare endocrine malignancy accounting for 0.005% of all cancers and <1% of primary hyperparathyroidism (7). Our patient has no history of exposure to external radiation or secondary and tertiary HPT due to chronic renal failure, which has been described as the main risk factor; whereas he suffered mild vitamin D deficiency, which may represent a trigger for hyperparathyroidism (8).

Although PC mostly occurs as a sporadic disorder, some hereditary syndromes associated with PC may be found (9). They include the HPT-JT syndrome, MEN syndromes, and potentially the non‐syndromic familial isolated primary hyperparathyroidism (FIHP) (10). Genomic alterations identified in PC are mostly represented by CDC73 germline gene mutations, codifying for a loss-of-function protein termed parafibromin. Moreover, whole-exome sequencing of PC has been recently performed, identifying mutations in other genes, such as mTOR, KMT2D, CDKN2C, THRAP3, PIK3CA, and EZH2 genes, as well as CCND1 gene amplification. Particularly, alterations in the PI3K/AKT/mTOR pathway can usually be found in sporadic forms (11). Unfortunately, in the case described here, the patient had no family history of hyperparathyroidism and refused genetic analysis.

In the pediatric population, PC, which can be associated with features of rickets, represents an exceptional event, considering that less than 20 cases have been reported in the literature, with a median age of presentation of 13 years. Based on the small number of cases, at a young age genetic evaluation should be considered. As for adults, no standardized clinical management has been developed, but surgery appears the best approach (12).

Since PC represents an extremely rare tumor, diagnosis strictly relies on histological criteria. Nevertheless, some clinical features may be useful to predict malignancy. High levels of calcium (> 14 mg/dl) and PTH (> 800 pg/ml or more than 10 times the upper normal value), associated with a single enlarged gland (> 3 cm) or signs of adjacent soft tissue invasion at sonography, should guide the clinician to search for carcinoma.

In the presented case, as in other cases described by literature, nuclear medicine imaging techniques, such as MIBI scan and ^18^F-FDG PET-CT, were helpful in localizing the mass and predicting malignancy, respectively. Moreover, ^11^C-methionine PET-CT and ^18^F-Fluorocholine PET-CT may also be used to locate hyperfunctional parathyroid in PC, especially in cases of relapse of the disease or in the occurrence of ectopia (13). Indeed, as for benign parathyroid lesions, PC may potentially develop in several anatomic locations including the mediastinum, the retro- or para-esophageal space, intrathymic or intrathyroidal sites, as well as the carotid sheath (14). The wide variety of possible ectopic locations has been recently confirmed by an unusual case of intrapericardial PC, pointed through 99Tc-Sestamibi Scan and MRI and successfully treated with radical surgery (15).

As recently confirmed, when PA localization is unusual or inconclusive despite extensive imaging, PTH measurements using needle washout fluid and immunocytochemistry with GATA-3 antibody for PA-suspected nodules may be a very useful tool (16). Otherwise, according to the guidelines of the American Association of Endocrine Surgeons (17), preoperative parathyroid FNA is not recommended if PC is suspected and was not performed in our case.

Histological examination can distinguish between PC, PA, or an atypical parathyroid adenoma (APA), based on the presence of specific features, such as capsular invasion with growth into adjacent tissues, vascular or perineural tumor invasion, and metastasis. Moreover, PC is characterized by the presence of four or more associated features of malignancy, such as capsular invasion, more than five mitoses on 10 hpf, intratumoral fibrous bands, tumor necrosis, small cells with high nucleus, cytoplasmic ratio, diffuse cellular atypia, presence of macronuclei; whereas the presence of only one to three of these features, qualifies for a diagnosis of APA (18). Furthermore, immunohistochemistry may play a crucial role in the differential diagnosis. Although in the reported case it was not assessed, parafibromin staining may play a prognostic role in patients with PC. Indeed, as widely described by a recent review (19), negative immunohistochemical staining of parafibromin seems to be promising in predicting outcomes for patients with PC, indicating a higher risk of recurrence/metastasis and mortality. Recent findings showed that cancer‐derived immunoglobulin G (CIgG) is expressed in parathyroid neoplasms and highly expressed in PC. Compared to immunohistochemical staining for parafibromin, CIgG showed favourable diagnostic accuracy for differentiating PC from PA or PH, resulting to be an independent prognostic factor for relapse prediction in PC too (20).

### 3.2 Treatment and Follow-Up

Radical surgery is the first-choice treatment in PC and is the most effective therapy to control hypercalcemia and other clinical manifestations. In the reported case, the clinical presentation oriented to perform en bloc surgery based on ipsilateral thyroid loboistmectomy and central lymph nodes dissection, as indicated in case of carcinoma (21). Since PC shows high rates of recurrence, ipsilateral completion surgery, if not already performed, should be considered when malignancy is confirmed. Moreover, since non-operative therapy failed to show a significant effect on the course of disease, surgery actually represents the first-choice treatment even in disease relapse or metastasis, if possible. When not, some local therapies, such as radiofrequency ablation, arterial embolization, or alcohol injection may be evaluated alone or in combination in order to improve serum PTH or calcium levels (22). On the other hand, although some anecdotal reports of short-term remission after chemotherapy or radiotherapy have been documented, adjuvant treatment appears not to be satisfactory in terms of recurrence rates and overall survival and no standard protocol has been developed, to date (23). An exceptional case of effective synergistic action of combined therapies is represented by a single patient with metastatic PC, who achieved long-term remission for 17 years after multiple lines of treatment (surgery, radiation, zoledronic acid, cinacalcet, and temozolomide) (24).

In clinical practice chemo- and radiotherapy seem to be reserved for selected cases of high-stage disease, when surgery or other local therapies failed or cannot be performed.

Moreover, tyrosine kinase inhibitors (TKIs) might play a key role in affecting mineral bone metabolism, as extensively studied and summarized by the Zannettino group (25). Indeed, they not only have shown efficacy in suppressing angiogenesis and proliferation but also seem to be effective in controlling hypercalcemia and inhibiting bone resorption. This inhibitory effect, potentially useful in improving the quality of life in patients affected by recurrent PC, may occur both through a direct action on osteoclasts and osteoblasts and an indirect action *via* several cellular pathways (26). For these reasons, TKIs, such as cabozantinib (27), sorafenib (28), and vandetanib (29), were given in a few case studies reported in the last few years, and in some selected cases the drug choice was also guided by patient-specific mutation analysis (29). In one patient with PC in HPT-JT syndrome with lung metastatic disease, the use of sorafenib brought normocalcemia and radiographic stability of neck and lung tumors. After 3 years of therapy, his calcium and parathyroid hormone levels started to rise again with disease progression in the lungs. Sorafenib was discontinued and a second more potent antiangiogenic inhibitor, lenvatinib, was initiated. After 20 months the disease was radiographically stable, and his calcium level was well controlled without any medication (30). When well-tolerated, TKIs ensure a radiographic and hormonal control of disease, associated with normalization of calcium, at least in the medium-short term. However, some patients had to discontinue the medication because of treatment‐related toxicities.

Among novel therapies, anti-PTH immunotherapy, biologic agents involved in Cyclin D1 pathway or acting on the PC immune microenvironment are being tested *in vitro* and may represent valid options in the future (31).

PC’s general prognosis and overall survival are connected to the stage at diagnosis. In the case of low stages and resectable disease, the prognosis remains acceptable. Nevertheless, the extreme variability in terms of rate and time of recurrences recommends a life-long follow-up, based on serum calcium and PTH levels and ultrasound surveillance (32).

An explanatory case of the unpredictability of this clinical entity has recently been reported. A 53-year-old woman, after a first lower right parathyroidectomy in 1993, experienced multiple relapses both in terms of hypercalcemic crisis and radiological recurrences, which required several surgeries, associated with radiotherapy and medical treatment, before reaching the histopathological criteria for the definitive diagnosis of PC (33).

### 3.3 Systemic Manifestations

PC is uncommon, but when it occurs, it is commonly associated with conditions strictly connected with primary hyperparathyroidism, such as severe hypercalcemia, osteoporosis, nephrolithiasis, and renal insufficiency. Furthermore, any other disease linked to hypercalcemia, malignant tumor, or both, may potentially occur. In the case described, we reported the extremely rare, almost unique, combination of several systemic manifestations, which are discussed below (**Table 2**).

**Table 2:** Main manifestations occurred in the discussed case of Parathyroid Carcinoma (PC).

Condition	Manifestation	Treatment
**Hypercalcemic Crisis**	Gastrointestinal, cardiac or neurological symptoms	Surgery of PC Fluids Diuretics Bisphosphonates Calcimimetics Calcitonin Steroids Hemodialysis
**Hungry Bone Syndrome**	Prolonged and severe hypocalcemia after parathyroid surgery	Calcium Vitamin D3 Bisphosphonates before surgery?
**Pancreatitis**	Nausea, Vomiting Abdominal Pain CT findings	Fluids Analgesics Antibiotics Surgery
**Venous Thrombosis**	Radiological findings	Anti-Coagulants
**Bone manifestations**	Osteitis fibrosa cystica Bone cysts Brown tumors Fragility fractures	Surgery of PC Antiresorptive drugs Orthopedic treatment
**Chondrocalcinosis and Synovitis**	Joint pain X-Ray and US findings	Immunosuppressors
**Neuropsychiatric symptoms**	Neuropathy Depression Anxiety Cognitive impairment	Surgery of PC Psychological support Neuroleptics

Possible clinical presentation and treatment are reported.

Possible clinical presentation and treatment are reported.

#### 3.3.1 Hypercalcemic Crisis

Severe hypercalcemia with gastrointestinal, cardiological, or neurological symptoms represents a medical emergency and is often associated with malignancy, even if it may also occur in adenoma or parathyroid hyperplasia. Anyway, aggressive fluid therapy is the first-line therapy to promote diuresis and reduce calcium levels. Waiting for surgery, diuretics, bisphosphonates, or calcimimetic drug, such as cinacalcet, may be helpful in stabilizing calcium levels and controlling symptoms. Based on its effect on the calcium-sensing receptor, cinacalcet was first used in 1998 specifically in the case of PC (34). Since then, it has proven useful, alone or in combination with other medications, in treating several cases of malignancy-associated hypercalcemia (35). Calcitonin, despite its rapid effect, is rarely used and carries the risk of tachyphylaxis and possible rebound hypercalcemia after 24 hours. Furthermore, steroid treatment is largely used in clinical practice to manage malignancy-related hypercalcemia; whereas low-calcium or calcium-free hemodialysis is the main rescue therapy, especially in case of severe acute renal failure (36). Although not approved for this indication, denosumab has proven useful in managing hypercalcemia caused by PC, as recently happened in the case of an 84-year-old female who developed refractory hypercalcemia after parathyroid surgery (37). Moreover, the combination of monthly denosumab with cinacalcet has been successfully used in the long-term management of parathyromatosis-related refractory hypercalcemia (38). Local approaches, such as radiofrequency ablation, may be helpful in lowering calcium levels when hypercalcemia is sustained by secondary lesions, as documented by the case report of 20 lung radiofrequency ablation sessions for 50 lung metastases from PC (39). Another interesting case is represented by a patient with metastatic PC whose hypercalcemic crisis was treated first with surgery and then radiofrequency ablation on lung metastases. She was also able to carry two pregnancies to term thanks to intensive medical treatment with cinacalcet (40).

#### 3.3.2 Hungry Bone Syndrome

As in the case described, severe and prolonged (more than 4 days) hypocalcemia in the period immediately after parathyroid surgery is a common event, particularly if high calcium and PTH pre-operative levels are present. Low calcium values can be associated with hypomagnesemia and hypophosphatemia and may persist for months or years after parathyroidectomy, requiring chronic calcium and alfacalcidol or calcitriol treatment, as occurred in our patient, who needed calcitriol replacement therapy for 8 months after surgery. In terms of preventing HBS, vitamin D3 replacement represents a useful tool, whereas preoperative treatment with bisphosphonates in HBS is controversial. A retrospective case series of patients preoperatively treated with zoledronate showed a low frequency of postoperative HBS (41). In contrast, other case reports documented that short-term preoperative bisphosphonate treatment may exacerbate postoperative hypocalcemia by reducing bone resorption, without allowing time for a coupled decrease in bone formation (42).

In our case, 4 mg zoledronate was preoperatively given with the primary aim to reduce the life-threatening calcium levels. As mentioned above, according to the current state of the art, it is difficult to define if the choice of a long-lasting bisphosphonate could have worsened the postoperative hypocalcemia. Certainly, in cases with a high risk of developing HBS, such as the reported one, who also presented multiple bone brown tumors and severe femoral and lumbar osteoporosis, a close follow-up strategy should be applied in order to establish the right timing for the introduction of a possible calcium and calcitriol supplementation therapy.

#### 3.3.3 Pancreatitis

Alcohol and biliary tract stones are the main causes of acute pancreatitis (AP), while AP due to primary hyperparathyroidism represents an uncommon event, an extremely rare one if hypercalcemia is supported by a PC (43). The etiologic relationship between these conditions was clearly documented in the past (44) and in some pathophysiological mechanisms have recently been proposed: pancreatic duct obstruction by deposition of calcium; autodigestion of the pancreas due to trypsinogen to trypsin corversion, triggered by high calcium levels; genetic variants, such as SPINK 1 (serine protease inhibitor Kazal type 1) and CFTR (cystic fibrosis transmembrane conductance regulator) mutations (45). Since patients with hypercalcemic crises show a higher prevalence of pancreatitis, surgical or medical treatment of primary hyperparathyroidism should always be considered. In addition, pancreatitis can be treated conservatively with intravenous fluids, analgesia, and broad-spectrum antibiotics, as occurred to our patient, or with surgical necrosectomy, according to the severity of the disease. Furthermore, both exocrine and endocrine pancreatic insufficiency may occur after the event, treatable with appropriate therapies. In conclusion, the unusual detection of hypercalcemia in a patient with AP should always alert physicians to the presence of primary hyperparathyroidism, even sustained by a PC. An explanatory case is represented by a patient with multiple ectopic parathyroid carcinomas, including a giant one in the anterosuperior mediastinum, who underwent surgery three different times. The persistence of disease was detected since the patient suffered recurrent AP associated with hypercalcemic crisis insensitive to calcium-lowering agents (46). On the other hand, the case of AP in PC that occurred during pregnancy still has historical value (47).

#### 3.3.4 Venous Thrombosis

Since malignancy increases the risk of thrombosis more than four-folds, venal thrombosis can be frequently found in paraneoplastic syndrome (48). Furthermore, hypercalcemia in primary hyperparathyroidism may contribute to inducing a hypercoagulable state, although the pathogenetic mechanisms are not fully well known. Hypercalcemia *per se* can induce a hypercoagulable state according to studies in animal models (49), while dehydration and hemoconcentration associated with gastrointestinal manifestations of hypercalcemic crisis have been proposed as potential additional factors for the development of thromboembolism (50). LMWH represents the first-line initial treatment for a patient with venal thrombosis in cancer, while unfractionated heparin (UFH) is recommended for patients with renal dysfunction (51). For this reason, UFH may be the choice treatment for venous thromboembolism (VTE) associated with parathyroid carcinoma, since most patients develop renal impairment. Fortunately, in the case described, the patient did not suffer from renal insufficiency so LMWH therapy could be administered. On the other hand, fondaparinux and direct oral anticoagulants (DOACs) for initial treatment of acute paraneoplastic VTE have insufficient data for the routine recommendation, while they can be used in chronic therapy (3-6 months or more in the case of active malignancy) (52). Patients with TVE are often asymptomatic and contrast-enhanced CT represents the best diagnostic and prognostic tool, also useful in orienting clinicians to the suspect of malignancy, leading them to perform radical surgery before the histological diagnosis of parathyroid carcinoma, as occurred in our case.

#### 3.3.5 Bone Manifestations

The catabolic action of PTH in parathyroid carcinoma brings consistent mineral bone density reduction, leading to osteitis fibrosa cystica, characterized by several manifestations, such as bone cysts, brown tumors, and increased incidence of fragility fracture. For this reason, it is always worth performing a DXA scan, in order to evaluate the presence of osteoporosis and the associated risk of fractures, which represents a common event in patients with severe primary hyperparathyroidism. On the other hand, brown tumors are today a rare condition, since they are associated with diagnostic delay. When carcinoma is suspected, the radiographic differential diagnosis with malignant bone lesion may be tricky. Indeed, as reported in our case, brown tumors can show a glucose uptake at 18-FDG PET and can easily be misdiagnosed as giant cell tumors at histological examination (53, 54). For these reasons, biochemical documentation of primary hyperparathyroidism results is essential for final diagnosis, avoiding surgery or other treatment usually reserved for metastatic lesions. Surgical treatment and consequent resolution of hyperparathyroidism may lead to an improvement of bone manifestations; if not enough, osteoporosis may be treated with antiresorptive drugs, whereas brown tumors may benefit from orthopedic treatment only in the case of large bone defects with spontaneous fracture risk or increasing pain (55).

#### 3.3.6 Chondrocalcinosis and Synovitis

The association between chondrocalcinosis and primary hyperparathyroidism is a common event, widely described in the literature. Indeed, chondrocalcinosis represents the main rheumatological manifestation, unusually associated with acute or chronic synovitis. Once chondrocalcinosis or synovitis have developed, their course seems to be independent from parathyroid function, so that it can continue even after surgery (56). For that, these conditions should be treated with specific immunosuppressants or immunomodulatory drugs. Colchicine has recently been successfully used to treat a deep vein thrombosis in Behçet disease, having a therapeutic rationale to reduce the inflammatory state at the endothelial level (57).

#### 3.3.7 Neuropsychiatric Symptoms

Literature documented a high prevalence of neuropsychiatric symptoms, such as depression, anxiety, and cognitive impairment, in patients affected by primary hyperparathyroidism, especially with severe hypercalcemia. As for the case described, the diagnosis of carcinoma may contribute to worsening mood deflection and panic. Even if there is no specific indication for surgery for these manifestations, patients usually experience a substantial improvement after normalisation of calcium values, as occurred in our case, after parathyroidectomy or medical treatment (58). Finally, only two other cases of sensory-motor peripheral polyneuropathy associated with primary hyperparathyroidism in parathyroid carcinoma have already been described (59, 60), while an association between benign PHPT and peripheral neurological alterations has been documented (61). However, in our case of parathyroid carcinoma, as for the two mentioned above, the presence of a malignant tumor may have played a role in the development of neuropathy in terms of paraneoplastic syndrome. Radical surgery once more represents the first-choice treatment to improve neurological symptoms.

## 4 Conclusions

The diagnosis of PC can prove to be an extremely difficult challenge, for which it is always advisable to integrate clinical, biochemical, and radiological elements, as well as a careful study of the associated systemic manifestations. In any case, in consideration of the remote frequency of the disease and the wide clinical variability, histological diagnosis is always essential.

We reported an unusual case of life-threatening PC, associated with several systemic manifestations. In particular, the intensity of the symptoms and the evidence of multiple clinical manifestations helped us in the early suspicion of malignancy. Moreover, some radiological features, such as the size of the lesion, the evidence of soft tissue invasion at ultrasound imaging, and the intense glucose uptake at PET scan, have also proved useful in orienting towards radical surgery.

When feasible, en bloc surgery always represents the best treatment both in terms of disease control and to obtain an improvement of the associated clinical manifestations, especially if performed in a center dedicated to parathyroid surgery; on the other hand, non-surgical therapies should be reserved only for selected cases. Considering the absence of reliable data on the relapse rates over time, it is important to indefinitely continue surveillance through blood tests and ultrasound, while further imaging studies may be reserved for the clinical, biochemical, or radiological suspicion of recurrence. Additional studies, including those involving genetics, new drugs, or local approaches, are needed to define a standardized treatment program for PC.

Better knowledge of PC tumorigenesis and micro-environment is still required in order to establish a precision oncology approach, based on TKIs, immunotherapy, or potentially any other therapy targeted to pathogenetic pathways of PC. Moreover, the recent improvement in genomic profiling techniques may drive the development of even more personalized medications.

Further meta-analysis elaborations are needed to guarantee a systematic diagnostic and therapeutic approach. As recently recommended by International Collaboration on Cancer Reporting (ICCR) (62), it will be essential to establish standardized data reporting on this rare entity, in order to allow the output of a prognostic staging system.

In conclusion, considering that PC and its related conditions represent a clinical singularity, where limited resources are available to date, a multidisciplinary clinical approach is still pivotal for establishing the correct management both of tumor and systemic manifestations. A well-timed and radical surgery, performed in a high-volume center, represents the best therapeutic tool to ensure long-term disease control. As in the case reported, the simultaneous involvement of multiple specialists, such as an endocrinologist, surgeon, oncologist, radiologist, nuclear doctor, radiotherapist, gastroenterologist, hematologist, and rheumatologist, has allowed for establishment of a therapeutic approach as suitable as possible for the patient. At the time of writing, 8 months after surgery, both his clinical condition and quality of life have consistently improved, but further follow-up is needed to assess the evolution of the disease.

## Data Availability Statement

The raw data supporting the conclusions of this article will be made available by the authors, without undue reservation upon reasonable request.

## Ethics Statement

Written informed consent was obtained from the individual(s) for the publication of any potentially identifiable images or data included in this article.

## Author Contributions

LZe, PL, CAR, CP, AC, DER, VR, LZa, and MR analyzed and interpreted the patient data. LZe, PL, and AP were major contributors in writing the manuscript. All authors contributed to the article and approved the submitted version.

## Conflict of Interest

The authors declare that the research was conducted in the absence of any commercial or financial relationships that could be construed as a potential conflict of interest.

## Publisher’s Note

All claims expressed in this article are solely those of the authors and do not necessarily represent those of their affiliated organizations, or those of the publisher, the editors and the reviewers. Any product that may be evaluated in this article, or claim that may be made by its manufacturer, is not guaranteed or endorsed by the publisher.
